# YAP/TAZ in Bone and Cartilage Biology

**DOI:** 10.3389/fcell.2021.788773

**Published:** 2022-01-04

**Authors:** Mylène Zarka, Eric Haÿ, Martine Cohen-Solal

**Affiliations:** ^1^ INSERM UMR 1132 BIOSCAR, Hôpital Lariboisière, Paris, France; ^2^ Faculté de Santé, Université de Paris, Paris, France

**Keywords:** osteocyte, bone, cartilage, YAP, TAZ, hippo signaling, biomechanic, mechanotransduction

## Abstract

YAP and TAZ were initially described as the main regulators of organ growth during development and more recently implicated in bone biology. YAP and TAZ are regulated by mechanical and cytoskeletal cues that lead to the control of cell fate in response to the cellular microenvironment. The mechanical component represents a major signal for bone tissue adaptation and remodelling, so YAP/TAZ contributes significantly in bone and cartilage homeostasis. Recently, mice and cellular models have been developed to investigate the precise roles of YAP/TAZ in bone and cartilage cells, and which appear to be crucial. This review provides an overview of YAP/TAZ regulation and function, notably providing new insights into the role of YAP/TAZ in bone biology.

## 1 Introduction

YAP (*yes associated protein*) and TAZ (*transcriptional coactivator with PDZ-binding motif*) were identified in mammals in 1995, and 2000, respectively (Sudol et al., 1995; Kanai et al., 2000). YAP/TAZ are transcriptional cofactors considered important cellular mediators that define the cell fate, such as differentiation, proliferation or apoptosis. Because of this central role, YAP/TAZ regulate numerous physiological cellular processes and thereby act as major protagonists in the maintenance of tissue homeostasis but also represent a target in different pathological contexts.

Bone and cartilage are two tissues particularly regulated by mechanical cues because tissue adaptation and remodelling in response to loading are essential to maintain their integrity. Dysregulation of this mechanoadaptive mechanism leads to osteoarticular pathogenesis such as osteoporosis or osteoarthritis. Hence, YAP/TAZ signaling may represent a central mediator that maintains constant adaptation of bone and cartilage tissues in response to modification of the mechanical environment. For this reason, numerous recent studies have aimed to improve our knowledge of YAP/TAZ regulation in bone and cartilage.

## 2 Bone and Cartilage

### 2.1 Bone Biology

**FIGURE 1:**
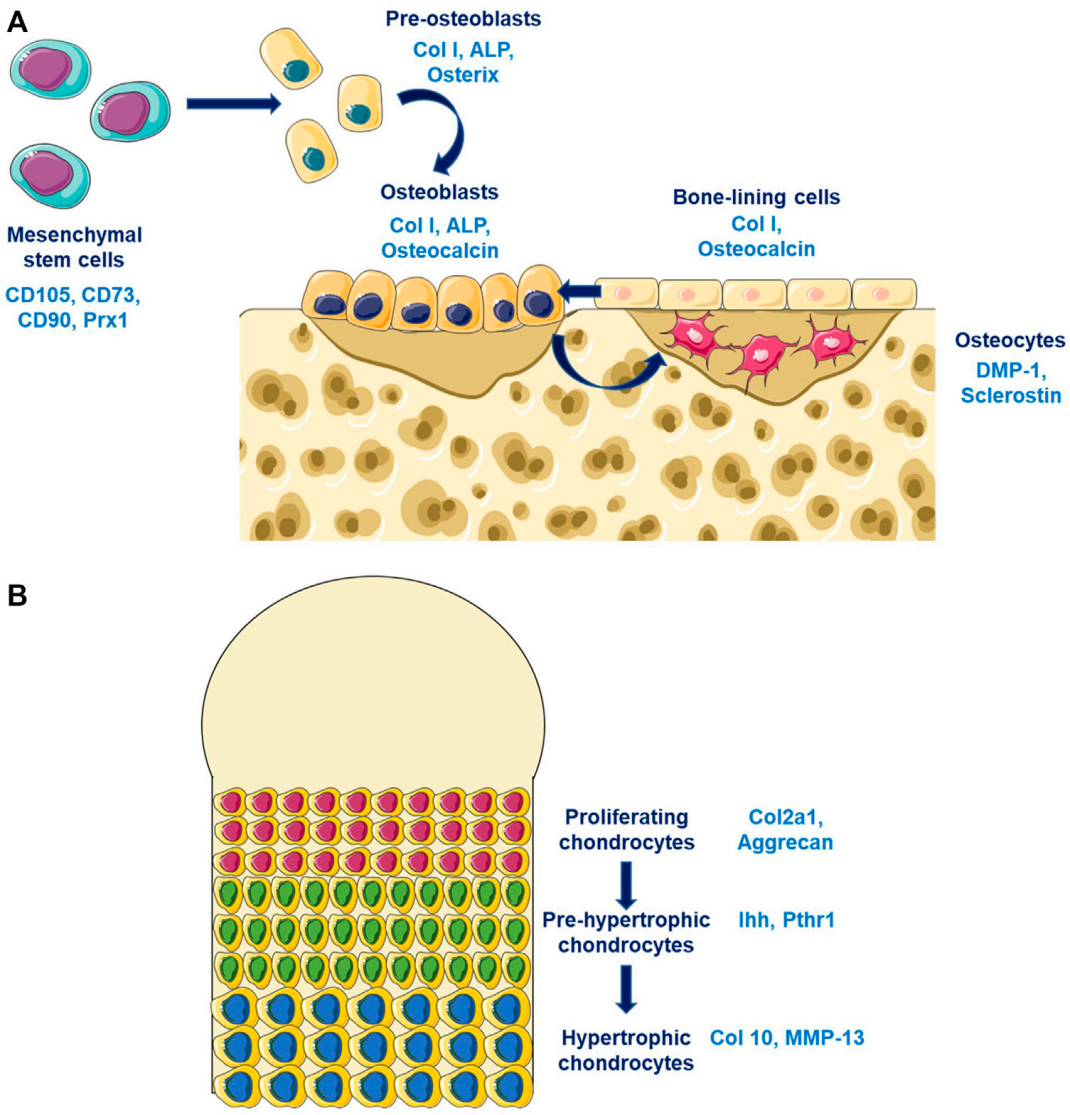
Bone and cartilage cells differentiation **(A)** Osteoblasts differentiation are characterized by the expression of different markers according to the stage of differentiation. Pre-osteoblasts expressed early osteoblastic genes such as Osterix while old osteoblasts/osteocytes expressed late osteoblastic genes such as DMP-1. **(B)** Chondrocytes differentiates from proliferating chondrocytes expressing Col2a1 in the surface layer through an hypertrophic phenotype characterized by the expression of late markers such as Col10.

Bone is a dynamic tissue characterized by a permanent remodeling allowing adaptation to mechanical environment. Bone integrity is maintained by its composition, its quality and its quantity. These characteristics are tightly regulated by different soluble factors whose actions is finely coordinated spatially and temporally by bone cells (osteoblasts, osteoclasts, and osteocytes). The major component of bone is the extracellular matrix that is composed principally by the collagen type I. This matrix was synthetised by osteoblasts that come from the differentiation of mesenchymal stem cells that expressed Prx1 (Figure 1A). Young osteoblasts are characterised by the expression of early markers such as the transcription factors Osterix (Osx), while more matures osteoblasts expressed the Osteocalcin (Ocn). The osteoblasts differentiation is regulated by different factors notably Runx2 and principally by the Wnt/β-catenin pathway. Osteocytes represents the ultimate stage of differentiation for osteoblasts that have been included in the bone matrix during the process of bone mineralization. Late osteoblasts/osteocytes expressed late markers such as the Dentin Matrix Protein 1 (DMP1). The third bone cell types are the osteoclasts that was originated from the hematopoietic stem cells lineage and that is responsible for bone degradation. Three mains soluble factors are essential for osteoclastogenesis, the RANK-L, the M-CSF, and Osteoprotegerin (OPG). These factors are particularly important for the coupling of osteoblasts and osteoclasts during the bone remodeling process. The renewal of bone matrix is allowed by bone remodeling which is divided in different phases: 1) the initiation of bone remodeling; 2) the bone resorption; 3) the bone formation; and 4) the matrix mineralisation. The coordinated action of osteoblasts and osteoclasts in time and space are partly regulated by the RANK-L/OPG. RANK-L, and its antagonists OPG, are two ligands synthetised by osteoblasts, and whose expression is modulated by the Wnt/β-catenin pathway in order to maintain a balanced between formation and resorption.

The bone remodeling is regulated by systemic factors principally hormones such as oestrogen and parathormone, but also by growth factors that are included in the bone matrix or by cytokines synthetized locally. TGF-β and BMPs are released by bone matrix during resorption and allow the regulation of osteoblastogenesis and osteoclastogenesis. TGF-β are able to induced the recruitment and the proliferation of osteoclasts and osteoblasts precursors. It also regulates the expression of RANK-L/OPG by osteoblasts and inhibits terminal differentiation of osteoblasts. BMPs could also regulates osteoblastogenesis by inducing the expression of Osx and Runx2, or two antagonists of the Wnt/β-catenin pathway, Dkk1, and sclerostin.

### 2.2 Cartilage Homeostasis

Cartilage is an avascular tissue composed by chondrocytes and extracellular matrix. Cartilage matrix is composed mostly by the collagen type II and by proteoglycans which allow their mechanical properties. The extracellular matrix protects chondrocytes from mechanical loading, represents a storage area for cytokines and growth factors, and controls nutriments diffusion and contribute to the transmission of extracellular signals to chondrocytes. Chondrocytes at the cartilage surfaces are characterized by a strong expression of the collagene type II, while hypertrophic chondrocytes in the mineralized compartment are expressing the collagen type X (Figure 1B). The early stage of chondrocytes differentiation is mainly modulated by the Sox9 transcription factors that regulates the collagen type II expression, while the late stage of hypertrophic chondrocytes is mostly controls by Runx2. Different signaling pathway are essential for chondrogenesis such as the Wnt/β-catenin pathway, the TGF-β/BMPs pathway, and the sonic hedgehog pathway (Shh). TGF-β stimulates the chondrocytes at early stage of differentiation while it inhibits terminal differentiation. The role of the canonical Wnt/β-catenin pathway in chondrocytes is complex since it could inhibit chondrogenesis and stimulates chondrocytes hypertrophy.

## 3 YAP and TAZ Biology

The YAP/TAZ complex is a downstream effector of the Hippo signaling pathway, discovered in *Drosophila* and described as a main regulator of organ growth during development (Dong et al., 2007; Pan, 2007). Several studies identified the main actors of Hippo signaling in *Drosophila*: Warts (Justice et al., 1995; Xu et al., 1995), Salvador (Kango-Singh et al., 2002; Tapon et al., 2002), Hippo (Harvey et al., 2003; Jia et al., 2003; Pantalacci et al., 2003; Udan et al., 2003; Wu et al., 2003), and Mats (Lai et al., 2005). The mutation of each of these proteins leads to a hyper-proliferative phenotype that allowed for identifying Hippo signaling as a regulator of tissue homeostasis. Yorki, the YAP/TAZ ortholog, identified in 2005 as a downstream effector of this pathway and can negatively regulate apoptosis, and induce cellular proliferation (Huang et al., 2005). The discovery and functional description of Hippo signaling in *Drosophila* allowed for considerable progress in understanding the mechanisms in mammals.

### 3.1 YAP/TAZ Functions

YAP/TAZ functions are numerous and are coordinately fine-tuned at the cellular and nuclear level. Mostly, YAP/TAZ are transcriptional co-factors acting directly on their target genes via their co-factors, and notably TEAD family members. YAP/TAZ are also signaling molecules implicated in the communication between Hippo signaling and other signaling pathways.

#### 3.1.1 Transcriptional Co-activation

YAP/TAZ are transcriptional co-activators without a DNA binding domain and therefore require interaction with molecular partners. This interaction allows for the expression of target genes that control proliferation, growth, and cell survival. Among those genes, the most described are CYR61, CTGF, ANKRD1, REG, AXL, and MYC. Several transcription factors have been described to interact with YAP/TAZ, mostly members of the TEAD family. This family consists of four homologous transcription factors, TEAD1-4, and which share the same structural domain (Kaneko and DePamphilis, 1998). The TEAD family facilitates the tumorigenic effect induced by YAP *in vivo* and induces gene expression required for proliferation and cellular growth (Zhao et al., 2008; Zhao et al., 2009; Liu-chittenden et al., 2012). The expression of some TEAD family members is strongly increased in a large number of cancer types and so could be used as prognosis markers of disease progression (Zhou et al., 2016). Finally, YAP/TAZ can interact with other transcriptional cofactors such as p73, the RUNX family and SMAD to induce apoptosis, and differentiation or proliferation (Kim et al., 2018).

#### 3.1.2 Interaction of YAP/TAZ With Others Signaling Pathways

**FIGURE 2:**
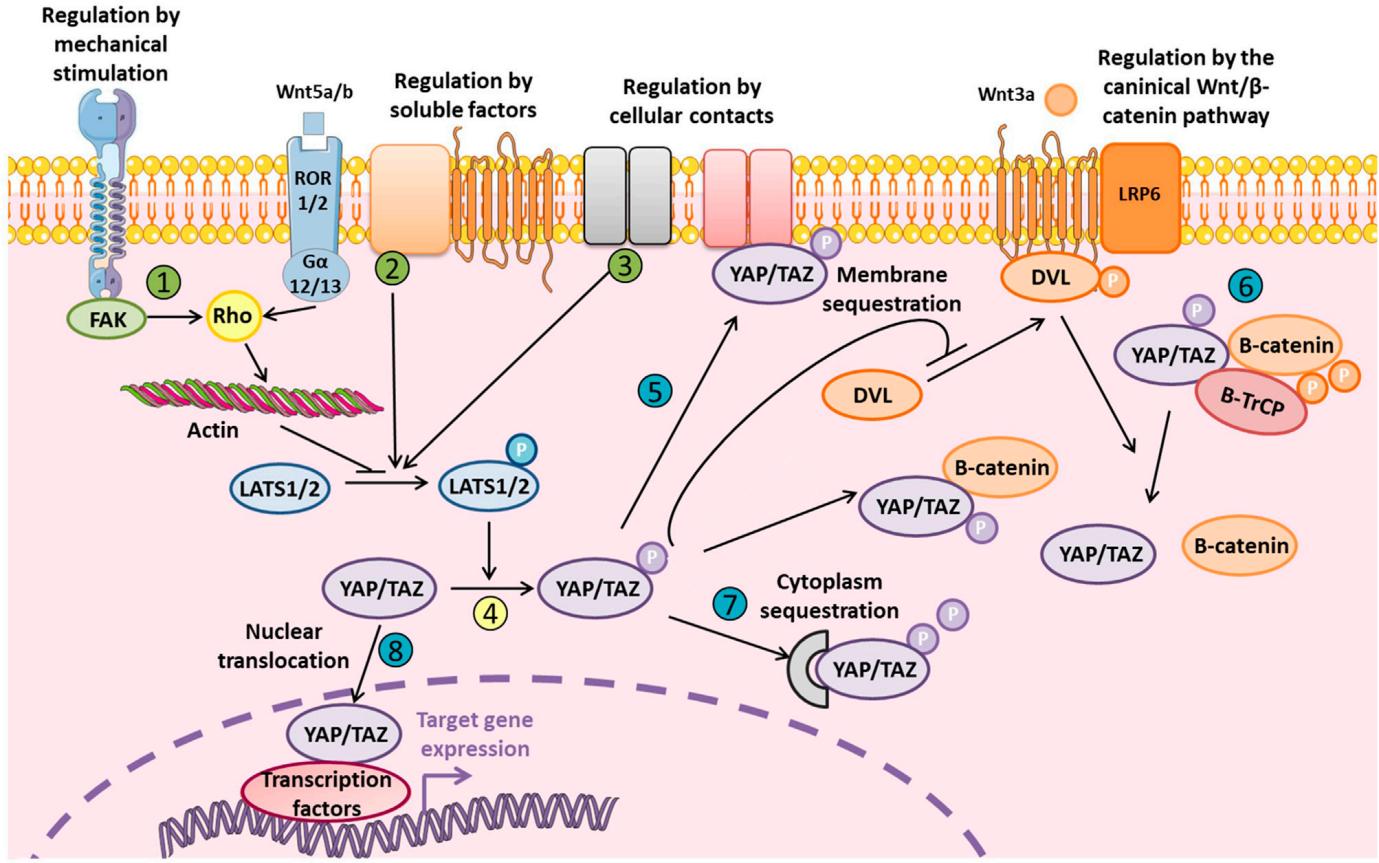
Levels of regulation of YAP and TAZ YAP/TAZ regulation is fine-tuned and mediated by external stimuli or soluble factors via LATS1/2 (in green: 1, 2, and 3) that phosphorylates YAP/TAZ (in yellow: 4). YAP/TAZ localization and degradation could be also modulated at different levels (in blue: 5, 6, 7, and 8). Phosphorylated inactive YAP/TAZ can induce cytoplasmic sequestration of β-catenin and inhibit Wnt/β-catenin signaling by inhibiting Dvl translocation to LRP5/6-Frizzled complex. Phosphorylated YAP/TAZ are also present in the β-catenin proteosomal degradation complex. YAP/TAZ could also be a transcriptional co-factor for β-catenin and its interaction with TCF/LEF. Finally, non-canonical Wnt/β-catenin signaling inhibits YAP/TAZ phosphorylation via LATS1/2.

YAP/TAZ interacts with different signaling pathways such as the Notch, Wnt/β-catenin, TGF-β, and BMP pathways. The Wnt/β-catenin pathway, which is crucial for osteoblastogenesis, is closely related to YAP/TAZ, and Hippo signaling (Figure 2). Therefore, inhibiting YAP/TAZ via Hippo signaling could represent a negative regulation of the Wnt canonical pathway. Indeed, the phosphorylation of YAP/TAZ inhibits the phosphorylation of Dvl by CK1δ/ε and subsequently the binding between Dvl, and LRP5/6-Frizzled induced by Wnt (Varelas et al., 2010a). Also, the Wnt ligand could activate YAP/TAZ via the non-canonical Wnt pathway by the FZD/G_α12/13_/Rho axis to induce target genes such as DKK1, BMP4, and IGFBP4 (Park et al., 2015). Different studies demonstrated a direct interaction between β-catenin and YAP in the transcriptional complex β-catenin/TCF4/YAP (Heallen et al., 2011; Konsavage et al., 2012; Deng F. et al., 2018). Finally, YAP/TAZ could be degraded into the cytoplasm by the proteasome whereby YAP/TAZ interacts with β-catenin to allow for binding to the ubiquitin ligase β-TrCP (Imajo et al., 2012; Azzolin et al., 2014).

Many studies also demonstrated that YAP/TAZ could interact with Smads signaling, mainly TGF-β and BMP signaling, and implicated in osteoblastogenesis (Chen et al., 2012; Moon et al., 2016). Indeed, YAP/TAZ are required for the TGF-β response by interacting with phospho-Smad2/3 to translocate into the nucleus (Hiemer et al., 2014; Mahoney et al., 2014). In response to high cellular density, phosphorylated YAP/TAZ could be retained in cytoplasm with Smad 2/3, and which inhibits the TGF-β response (Varelas et al., 2010b). Finally, YAP and TAZ act in synergy after BMP stimulation because YAP interacts with Smad1/5 to induce target genes, and whereas TAZ induces BMP4 expression (Alarcón et al., 2009; Lai and Yang, 2013).

### 3.2 YAP/TAZ Regulation

YAP/TAZ regulation is tightly modulated and occurs at multiple levels, notably by YAP/TAZ inactivation via phosphorylation leading to their degradation and/or cytoplasmic sequestration to avoid YAP/TAZ nuclear translocation (Figure 2). YAP/TAZ regulation could depend on Hippo signaling or be independent, via direct modulation of YAP/TAZ cellular localization. The cellular microenvironment is really important to take in consideration since it could restrain YAP/TAZ to nucleus or the cytoplasm, which can totally modify the modality of YAP/TAZ molecular regulation. In fact, for example, large surface area, and stiff matrix or the presence of mechanical forces lead to the nuclear translocation of YAP/TAZ. *In vitro* study which are performed on stiff plastic support, is associated with a basal activation state of YAP/TAZ due to the stiff properties of the matrix.

#### 3.2.1 YAP/TAZ Regulation via Hippo signaling

Hippo signaling pathway regulates a number of biological processes such as cellular proliferation, cell fate, cellular differentiation, organ size, and tissue homeostasis. The pathway is composed of a complex cascade of serine/threonine-protein kinase including the Hippo kinase core that consists of two enzymatic complexes, LATS1/2-MOB1A/B and MST1/2-SAV1. The kinase MST1/2, associated with its regulatory protein SAV, activates LATS1/2-MOB1A/B via phosphorylation (Chan et al., 2005; Praskova et al., 2008). This active complex can phosphorylate YAP/TAZ, with effects depending on the targeted serine (Zhao et al., 2010). YAP/TAZ phosphorylation induces the cytoplasmic sequestration, nuclear exclusion, and/or proteasomal degradation (Zhao et al., 2007, 2010; Lei et al., 2008; Liu et al., 2010). Among the different sites of phosphorylation, Ser127 (Ser89 for TAZ), and Ser381 (Ser311 for TAZ) are the most decisive for protein inactivation. In fact, the phosphorylation of Ser381 by LATS1/2 allowed for recruitment of the ubiquitin complex SCF^β–TRCP^E3 leading to YAP/TAZ degradation by the proteasome (Liu et al., 2010; Zhao et al., 2010). Moreover, Ser127 phosphorylation induced YAP/TAZ recognition by 14-3-3 protein and their cytoplasmic sequestration (Lei et al., 2008).

Regulation of LATS1/2 phosphorylation is an indirect regulation of YAP/TAZ activity and could be modulated by both soluble factors and/or cellular contact. Many soluble extracellular factors can regulate YAP/TAZ via Hippo signaling to promote cellular migration and proliferation. Members of the epidermal growth factor family, lysophosphatidic acid or sphingosine-1-phosphate, can inhibit LATS1/2, and subsequent YAP/TAZ nuclear translocation (Komuro et al., 2003; Omerovic et al., 2004; Fan et al., 2013; Reddy and Irvine, 2013; Haskins et al., 2014). G protein-coupled receptors can also modulate YAP/TAZ differentially depending on the subunit and ligand. Indeed, RCPGα12/13 are mostly activated by ligands such as LPA, and S1P or thrombin to activate YAP/TAZ dependent on Rho GTPase (Miller et al., 2012; Mo et al., 2012; Yu et al., 2012). However, G protein-coupled receptors associated with the Gα/s subunit can activate Hippo signaling via cAMP/protein kinase A signaling (Kim et al., 2013; Yu et al., 2013).

Cellular contacts can also facilitate the YAP/TAZ axis activation indirectly by modulating LATS1/2 activation. This activation involves three major complexes associated with the cellular membrane: NF2/KIBRA, SCRIB, and α-catenin/AMOT/AJUBA/NF2 (see Figure 2 from Totaro et al., 2018 and Meng et al., 2016 for review). KIBRA protein was identified upstream of Hippo signaling and can activate LATS1/2 (Baumgartner et al., 2010; Genevet et al., 2010; Yu et al., 2010). KIBRA can restrain proliferation notably on polarized cells because it negatively regulates YAP/TAZ via LATS1/2 phosphorylation (Xiao et al., 2011). Also in response to cellular polarity, SCRIB protein, described as a scaffold protein involved in this process, regulates Hippo signaling (Doggett et al., 2011; Verghese et al., 2012). For example, the SCRIB membrane delocalization observed during the epithelial–mesenchymal transition of cancer stem cells leads to YAP/TAZ activation by inhibiting Hippo signaling (Cordenonsi et al., 2011). The third major complex, α-catenin/AJUBA/NF2/AMOT, regulates cellular proliferation following adhesion, and cellular contacts. This situation may restrain YAP/TAZ activation via LATS1/2 phosphorylation in response to adherents junctions and cytoskeletal tension (Das Thakur et al., 2010; Kim et al., 2011; Rauskolb et al., 2014).

#### 3.2.2 Direct Regulation of YAP/TAZ Cellular Localization

YAP/TAZ regulation via membrane or cytoplasmic sequestration may occur by a distinct mechanism. YAP/TAZ sequestration at the cellular membrane that abolishes any transcriptional activity could be regulated by AMOT (Wang et al., 2011; Zhao et al., 2011). AMOT can also modulate YAP nuclear localization to facilitate its interaction with the transcriptional co-factor TEAD and promote YAP-dependant proliferation (Moleirinho et al., 2017). At adherents junctions, α-catenin interacts with YAP/TAZ/14-3-3 complexes to inhibit epidermal stem cell proliferation induced by nuclear translocation of YAP (Schlegelmilch et al., 2011). The WW domain of YAP/TAZ allows for direct interaction with PTPN14, which leads to YAP/TAZ cytoplasmic sequestration, and prevents their nuclear translocation (Liu et al., 2013; Michaloglou et al., 2013).

Cytoplasmic sequestration is not the only mechanism of YAP/TAZ inhibition independent of Hippo signaling because YAP/TAZ can also be inhibited at the nuclear level. This mechanism involves direct competition between YAP/TAZ and VGGL4 protein for fixation on the transcriptional cofactor TEAD (Jiao et al., 2014; Zhang et al., 2014).

### 3.3 Effect of the Cellular Microenvironment on YAP/TAZ

YAP/TAZ localization and activity are also regulated by different physical constraints that occur at the cellular level. These environmental constraints depend on matrix properties, the presence of a cellular contact or tension forces such as shear stress.

#### 3.3.1 YAP/TAZ Regulation via Cellular Junctions and Adhesion

Matrix rigidity and adhesion surfaces regulate YAP/TAZ localization and allow for modulation of cellular behaviours to adapt to the cellular microenvironment. Indeed, a stiff matrix or large adhesive area is associated with YAP/TAZ nuclear translocation (Dupont et al., 2011; Aragona et al., 2013; Totaro et al., 2017). Conversely, high cellular density inhibits YAP/TAZ translocation (Zhao et al., 2007; Wada et al., 2011; Hsiao et al., 2016). Therefore, YAP/TAZ are regulated by both the presence of a matrix contact that promotes their nuclear translocation and by the presence of a cellular contact that inhibits this process. In this context, integrin signaling is crucial for YAP/TAZ regulation by the organization of actin filaments and PI3K/PDK1 signaling, and which inhibits Hippo signaling. Indeed, the structural organization of the actin network and the formation of stress fibres are required to activate YAP/TAZ, independent of the ratio of G to F actin (Connelly et al., 2010; Dupont et al., 2011; Aragona et al., 2013). Hence, the inhibitors of actin polymerization and inhibitors of the actomyosin network reduce YAP/TAZ activity. This regulatory mechanism is independent of the Hippo pathway because LATS1/2 inhibition is not sufficient to restore YAP/TAZ activity in the presence of actin polymerization inhibitors (Dupont et al., 2011). In recent years, different studies have highlighted the integrin/FAK/CFC42/PP1A axis as a regulator of YAP/TAZ nuclear translocation (Elbediwy et al., 2016; Hu et al., 2017; Xiang et al., 2018). Notably, integrins β1 and α3 are upstream of YAP/TAZ activation in epithelial cells and transit-amplifying cells (Elbediwy et al., 2016; Hu et al., 2017). Also, integrin α5 controls osteoblast mechano-sensing and is required to induce YAP/TAZ nuclear translocation in osteoblasts under shear stress (Kaneko et al., 2014).

The activation of integrin and focal adhesion kinase by fibronectin stimulates PDK1 via PI3K to inhibit LATS1/2 and promote YAP/TAZ nuclear translocation (Kim and Gumbiner, 2015). All of these studies demonstrate that integrins are part of the cellular perception of the microenvironment and are thus capable of regulating YAP/TAZ.

#### 3.3.2 YAP/TAZ and Mechanical Forces

The YAP/TAZ regulatory mechanisms described above allow for the modulation of cellular responses to the different forces applied from the environment, notably shear stress. For example, mechanical stress regulates cellular proliferation, and as shown in quiescent epithelial cells. In these cells, stress activates the expression of anti-apoptotic genes (Birc5, AREG) as well as proliferative genes (c-Myc, Cyclin D1) via YAP and β-catenin nuclear translocation (Benham-pyle et al., 2015). Shear stress also regulates YAP/TAZ cellular localization, mainly described during atherosclerosis, and in which the hemodynamic environment regulates endothelial cells. Hence, the modification of shear stress induces an inflammatory response and the emergence of YAP/TAZ-dependant lesions, and homogenous shear stress inhibits this process (Wang K.-C. et al., 2016; Wang et al., 2016 L.). In zebrafish*,* YAP/TAZ activation resulted from actin filament reorganization in response to shear stress, and YAP/TAZ consecutive interaction with AMOT protein (Nakajima et al., 2017). Shear stress facilitates osteoblastogenesis from mesenchymal stem cells (MSCs) via RhoA activation and YAP/TAZ nuclear translocation (Kim et al., 2014).

It was also demonstrated *in vitro* that increasing the stiffness of a mineralized collagen glycosaminoglycan matrix allow osteoblastogenesis from bone marrow-derrived hMSCs through YAP/TAZ activation (Zhou et al., 2021). This result was elegantly confirmed during *in situ* bone regeneration in a bone defect model with a self-mineralizable matrix inducing osteoblastogenesis from MSC across time according to the level of mineralization (Li J. et al., 2021). It was demonstrated that stiffness could modulate YAP/TAZ through RAP2 downstream of the phospholipase Cγ1 (Meng et al., 2018). Indeed, at low stiffness, active RAP2 could act on LATS1/2 activation which lead to YAP/TAZ inhibition.

Interestingly, Major and its collaborators demonstrated that cellular volume should be more relevant than just stiffness of the matrix. Indeed, they shown opposite effect of stiffness in 2D vs 3D since 3D soft matrix favours osteoblastogenesis from adipose-derived stem cells (Major et al., 2019).

All of these results highlight the fact that all of forces emanating from the microenvironment are integrated at cellular level and affect YAP/TAZ activation states. In the context of bone, osteoblast lining cells and osteocyte matrix-embedded cells have different 3-dimensional mechanical environment which necessarily lead to a different regulation of YAP/TAZ.

In degenerative diseases, modifications of the matrix properties could also lead to the modification of YAP/TAZ activation that could contributes to the pathogenesis (Fearing et al., 2019). For example, in the adult nucleus pulposus (NP), cells are embedded in a soft matrix that becomes fibrotic and stiffness with age. Modifications of mechanical cues emanating from this altered matrix modify the cell shape and activate YAP that is normally sequestered in the cytoplasm.

## 4 YAP/TAZ and Bone Biology

Bone is a dynamic tissue associated with permanent remodelling that is required to adapt the bone structure and density to maintain physical integrity upon mechanical loading. Different studies have highlighted a role for YAP/TAZ in this process. Hence, YAP/TAZ regulates chondrogenesis and osteoblast differentiation from MSCs to late osteoblast stage/osteocytes. Recently, our lab and others have characterised the role of YAP/TAZ in osteocyte perilacunar/canalicular remodeling and in mechanotransduction.

### 4.1 Role of YAP/TAZ in Craniofacial and Dental Development

YAP/TAZ and the Hippo pathway are known to be implicated in development especially in organ size. Different works aims to elucidated their roles on craniofacial and dental development such as the work of Wang and its collaborators that demonstrates the role of YAP/TAZ in neural crest-derived craniofacial development (Wang et al., 2016a). Deletion of YAP/TAZ in cranial neural crest using Wnt1^Cre^ and Wnt1^Cre2SOR^ lead to embryonic lethality with vascular defect probably causing haemorrhage. This work indicates that YAP/TAZ regulate vascular development that is known to be essential for bone development. It was also demonstrated that YAP/TAZ modulates the secondary palate development notably by regulating genes involved in mineralization such as Phex, which could lead to the regulation of collagen cross-linking in the palate shelf mesenchyme (Goodwin et al., 2020). This suggest that YAP/TAZ could themselves influence their matrix stiffness by modulating gene implicated in bone mineralization.

YAP/TAZ was also study in the context of the generation of transit-amplifying cell (TAC) populations during growth of mouse incisor (Hu et al., 2017). It was shown *in vivo* that this process is modulated by the ITGA3-FAK-CDC42 signaling axis in order to activate YAP in a LATS-independent manner. This regulation led to nuclear accumulation of YAP and the maintenance of a high proliferation rate necessary to maintain organ renewal. Interestingly, Li and its collaborators demonstrates that the α-E catenin are able to inhibit YAP in the mouse incisor (Li et al., 2016). This regulation allows the establishment of non-dividing cells for dental mesenchymal condensation and epithelial invagination.

### 4.2 Role of YAP/TAZ in Bone and Cartilage Stem Cells Differentiation

**FIGURE 3:**
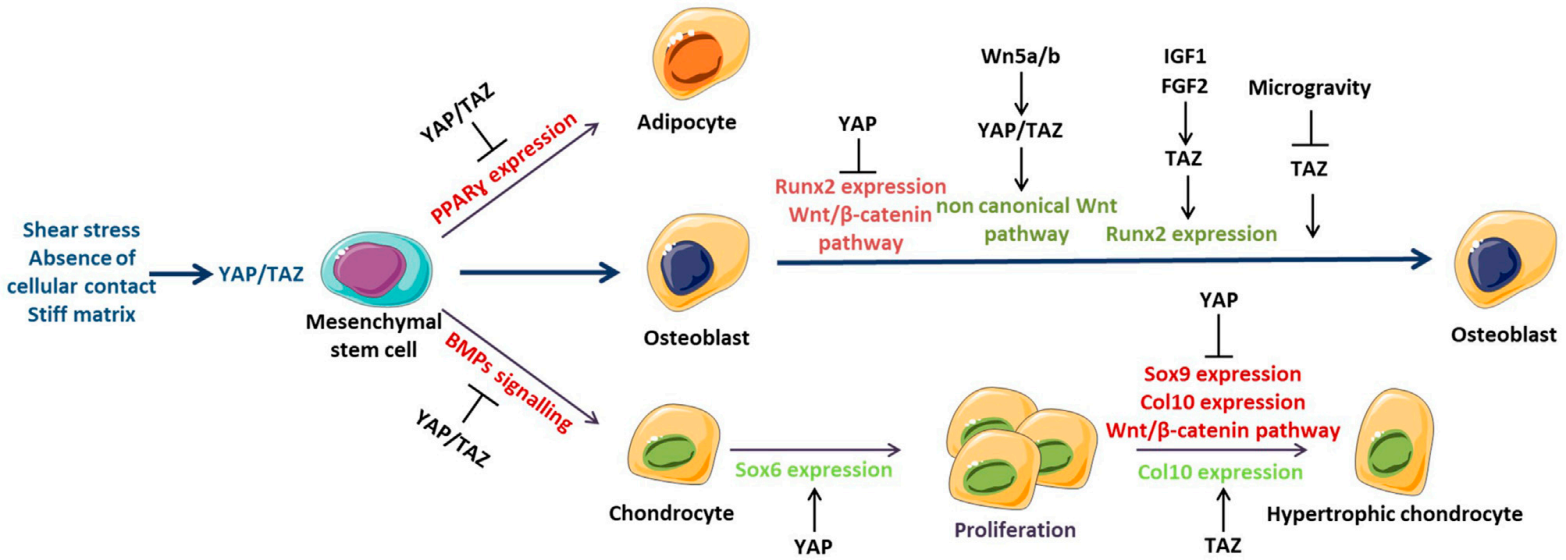
YAP/TAZ in bone and cartilage biology YAP/TAZ regulates mesenchymal stem cell commitment toward an osteoblastic lineage and inhibits adipogenesis and chondrogenesis by inhibiting PPARγ, and BMP, respectively. This process allows for osteoblast differentiation in relation to microenvironment modifications such as shear stress, absence of cellular contact, and/or a stiff matrix that promotes YAP/TAZ activation. YAP/TAZ regulate osteoblastogenesis by modulating Runx2 and the Wnt/β-catenin pathway.

YAP/TAZ allow for the MSC commitment toward an osteoblastic lineage while inhibiting adipogenesis and chondrogenesis (Figure 3) (Hong et al., 2005; Lorthongpanich et al., 2019). This differentiation process is regulated by different mechanisms such as matrix metalloproteinase (MMP) synthesis, cellular contact and shear stress. MSCs produce MT1-MMP (MMP-14) to induce matrix remodelling responsible for nuclear translocation of YAP/TAZ via the activation of integrin β1/RhoA axis *in vivo* (Tang et al., 2013). Thus, the matrix remodelling triggers the differentiation of MSCs toward an osteoblastic lineage rather than chondrogenesis or adipogenesis. In addition, the absence of cellular contact induces morphological changes in MSCs such as a large adhesion surface, thereby promoting osteoblastogenesis, and inhibiting adipogenesis (McBeath et al., 2004). Snail/Slug signaling also participates *in vivo* in the differentiation process via YAP/TAZ activation and subsequent expression of osteoblastic genes such as Runx2 (Tang and Weiss, 2017).

Dupont and collaborators demonstrated that the osteogenic differentiation normally induced in MSCs on stiff matrix was inhibited *in vitro* by YAP/TAZ small interfering RNA (siRNA) (Dupont et al., 2011). YAP/TAZ silencing enabled adipogenic differentiation even on a stiff matrix that inhibited this process, thus imitating a soft environment. Moreover, shear stress induced the nuclear translocation of YAP/TAZ and the differentiation of MSCs *in vitro* via the osteoblastic lineage associated with the increased expression of Runx2, Dlx5, and Msx2 (Zhong et al., 2013; Kim et al., 2014). This mechanism involves RhoA because RhoA inhibition abolished the nuclear translocation of YAP/TAZ and the concomitant to activate target genes.

Recently, a role of YAP/TAZ in subchondral bone stem/progenitor cells (SCP-SPC) was described *ex vivo* in the context of the radial extracorporeal shockwave (Zhao et al., 2021). They demonstrated that radial shockwave influences the self-renewal of SCB-SPC through modulation of YAP.

### 4.3 Role of YAP/TAZ in Osteoblastogenesis

Several studies have highlighted the role of YAP and TAZ in regulating osteoblastogenesis and their proteins partners (Table 1). Mostly, TAZ was described as a transcriptional co-activator interacting with Runx2, and the master regulator gene of osteoblastogenesis (Cui et al., 2003; Byun et al., 2014). The growth factors FGF-2 and IGF-1 were described *in vitro* as inducers of osteoblast differentiation by increasing TAZ expression and its nuclear translocation involving ERK signaling (Xue et al., 2013; Byun et al., 2014). Hence, the inhibition of TAZ by siRNA abolished osteogenic differentiation induced by FGF-2 and IGF-1 *in vitro* in cultures of rat bone marrow and C3H10 cell lines. In contrast to TAZ, YAP inhibited Runx2 activity in the osteoblast-like cells ROS 17/2.8 (Zaidi et al., 2004). Pan and collaborators demonstrated that YAP regulates osteoblastogenesis via Wnt/β-catenin signaling *in vitro* and *in vivo* (Pan et al., 2018). Finally, microgravity decreased the osteogenic differentiation induced by downregulation of TAZ activity on MSCs isolated from rat long bones (Chen et al., 2015). TAZ activation by lipophosphatidic acid blocked the inhibitory effect of gravity on osteoblast differentiation by inducing ROCK signaling.

**TABLE 1:** Partners of YAP/TAZ in bone and cartilage.

Bone partners	Cellular consequences	References
α-catenin	Cytoplamic retention of YAP to establish a group of non-dividing and specialized cells for formation of the tooth signalling centre, the enamel knot (EK)	Li et al. (2016)
Snail/Slug	Interaction inside the complex Snail/Slug-YAP/TAZ-Runx2 to stimulate MSC differentiation	Tang et al. (2016)
Runx2	Stimulate Osteocalcin gene expression and osteoblastogenesis	Cui et al. (2003), Hong et al. (2005)
PPARγ	Inhibit adipogenesis	Hong et al. (2005)
β-catenin	Stimulate osteoblastogenesis	Pan et al. (2018)
Smad1/5/8	Stimulate osteoblastogenesis in response to BMP-2	Wei et al. (2020)

YAP/TAZ, interacts in protein complex with different signaling pathway such as TGFβ/BMPs, and the Wnt/β-catenin pathway. YAP/TAZ, also interacts as DNA, and binding partners with Runx2.

Recently, mouse models were used to explore the role of YAP/TAZ in bone (Table 2). These works highlighted the differential role of YAP/TAZ according to stage of osteoblast differentiation. YAP/TAZ double knockout in the osteoprogenitors Osx+ or Prx1+ led to a lethality induced by ribcage malformation and the occurrence of haemorrhage, respectively, and during embryonic development (Kegelman et al., 2018; Xiong et al., 2018). The inducible double deletion of YAP/TAZ in Osx+ osteoprogenitors promoted osteoblastogenesis and bone formation in compact bone of 12-week-old mouse vertebrae. In parallel, a reduced mineral apposition rate resulted in the absence of any modified bone formation rate. Of note, YAP^fl/+^, and TAZ^fl/fl^; Prx1^Cre^ mice featured a bone mass owing to increased bone formation, and which suggests that YAP/TAZ have distinct roles depending on the stage of differentiation (Xiong et al., 2018). Thus, conditional deletion of YAP in fully differentiated osteoblasts from YAP^fl/fl^; Ocn^Cre^ mice resulted in bone loss associated with decreased osteoblast proliferation and differentiation. Moreover, bone marrow from YAP^fl/+^, TAZ^fl/fl^; Prx1^Cre^ and YAP^fl/fl^, TAZ^fl/fl^; Osx^Cre^ mice showed increased osteogenic differentiation, notably with increased levels of bone formation markers such as Osx, osteocalcin, and collagen I (Xiong et al., 2018). This double deletion in osteoprogenitors was associated with increased Wnt/β-catenin signaling and Runx2 expression. Hence, single deletion of YAP or TAZ in Osx + cells (YAP ^fl/fl^; Osx^Cre^, and TAZ^fl/fl^; Osx^Cre^) or double deletion of YAP/TAZ at the mature osteoblast/osteocyte stage (YAP^fl/fl^, TAZ^fl/fl^; DMP1^Cre^) decreased bone mass, which was associated with increased osteoclast activity, and decreased osteoblastogenesis (Kegelman et al., 2018; Xiong et al., 2018). YAP^fl/fl^, TAZ^fl/fl^; DMP1^Cre^ mice showed decreased osteoblast number and bone formation rate resulting from decreased mineralized surface and apposition mineral rate. Together, these data show that YAP/TAZ could promote the commitment toward an osteoblastic lineage but inhibit the activity of fully differentiated osteoblasts/osteocytes.

The role of each co-factor was also assessed. TAZ overexpression in osteoblasts or the administration of TAZ lentivirus in a model of bone loss promoted the increase in bone mass and density associated with increased levels of osteoblast markers such as Runx2 and osteocalcin (Yang et al., 2013; Zhang et al., 2016). Overexpression of TAZ in the osteoblast cell line C3H10 upregulated Runx2 transcriptional activity associated with increased TGF-β response and decreased Wnt-β-catenin signaling (Yang et al., 2013).

Finally, YAP/TAZ could contribute to bone fracture healing because YAP/TAZ deletion in adult mice impaired bone formation in the callus (Kegelman et al., 2021). Thus, YAP/TAZ accelerated bone fracture healing via the expansion and differentiation of periosteal osteoblast precursors.

### 4.4 YAP/TAZ and Osteocytes

Recent studies highlighted the implication of YAP/TAZ in osteocytes with a role in bone quality and adaptative mechanical features. Two roles of YAP/TAZ were described in osteocytes with specific functions in perilacunar/canalicular remodeling and in mechanotransduction. Kegelman and collaborators investigated the role of YAP/TAZ in osteocyte-mediated bone remodeling by the conditional deletion of YAP and TAZ in DMP1^Cre^ mice (Kegelman et al., 2020). The invalidation of YAP/TAZ in osteocytes resulted in lower bone mass and dysregulated matrix collagen content and organization, thereby reducing bone mechanical properties. The authors also showed that YAP/TAZ is crucial for TGF-β-induced matrix protease gene expression and osteocyte perilacunar/canalicular remodeling. In line with these findings, we assessed the implication of YAP/TAZ in osteocyte mechanotransduction and showed that YAP/TAZ translocated to the nucleus and activated their target genes in a 3D *in vitro* culture model of the MLO-Y4 osteocyte-like cell line under mechanical compression (Zarka et al., 2021). YAP/TAZ silencing by short hairpin RNA partially blocked the increased M-csf and Cxcl3 gene expression induced by osteocyte loading, which suggests their role as mediators of mechanically induced chemokine expression in MLO-Y4 osteocytes. Moreover, transcriptomic analysis of YAP/TAZ-deleted osteocytes under compression strain revealed the regulation of several factors that initiate the formation of dendrites. This observation suggests the central role of YAP/TAZ in the formation of a perilacunar/canalicular network and in osteocyte-mediated bone remodeling.

### 4.5 Role of YAP/TAZ in Chondrocyte Function

The involvement of YAP was mostly analysed in chondrogenesis given that YAP induces chondrocyte proliferation and inhibits their differentiation (Karystinou et al., 2015; Yang et al., 2017). Chondrocyte proliferation is controlled by YAP, which induces the expression of Sox6 required for the proliferation while inhibiting the expression of collagen type X, a marker of hypertrophic chondrocytes *in vitro* and *in vivo* (Deng et al., 2016). Hence, inhibition of YAP activity is necessary to allow chondrocyte differentiation because YAP inhibits the BMP response that is essential for chondrocyte differentiation *in vitro* (Karystinou et al., 2015). Also, YAP inhibits chondrocyte differentiation *in vitro* by reducing Wnt/β-catenin signaling, whereas chondrocyte de-differentiation was found associated with increased YAP/TAZ level induced by RhoA signaling (Yang et al., 2017). Consistently, YAP is mostly localised in the nucleus of pre-hypertrophic chondrocytes, and hypertrophic chondrocytes show decreased YAP nuclear localization during embryogenesis (Goto et al., 2018).

These data suggest that YAP/TAZ promote the commitment of chondrocyte differentiation while blocking the final hypertrophic differentiation as a compensatory mechanism. These was confirmed by a recent study demonstrating the role of TAZ during chondrogenesis *in vivo* (Li Y. et al., 2021). They show that TAZ expression increased during chondrogenic differentiation and that TAZ deletion using Col2a1^Cre^ mice inhibits growth plate and articular cartilage development. TAZ was found to promote chondroprogenitors cell proliferation while inhibiting chondrocyte maturation.

Overexpression of YAP/TAZ in chondrocytes induced by MOB1A/B and constitutive activation of YAP in cartilage resulted in a phenotype of chondrodysplasia (Table 3) (Goto et al., 2018; Vanyai et al., 2020). MOB1A/B deletion in mice revealed a low growth plate length and long bones, associated with altered proliferation, differentiation, and endochondral ossification. Primary chondrocytes isolated from these mice showed decreased proliferation related to decreased Sox9 expression induced by YAP/TAZ overexpression. In addition, Deng and collaborators demonstrated that YAP-specific overexpression in chondrocytes, in transgenic Col2a1-YAP mice or by Mst1/2 deletion under Cre-recombinase Col2a1, protected articular cartilage against osteoarthritis (Deng Y. et al., 2018). YAP overexpression attenuated NF-κB signaling and protected against extracellular matrix degradation by inhibiting matrix-degrading enzymes.

**TABLE 2:** Bone phenotype induced by YAP and TAZ modulation.

Genotype	Stage of differentiation	Bone structure	Histo-morphometric parameters	Age	References
YAP^fl/fl^; TAZ^fl/fl^; Prx1^Cre^	Mesenchymal stem cells	Lethality (severe hemorrhage and edema)	—	Embryonic lethality	Xiong et al. (2018)
YAP^fl/fl^; TAZ^fl/fl^; Osx^Cre^	Young osteoblasts	Lethality (neonatal asphyxiation due to ribcage malformation, fracture)	—	Neonatal lethality	Kegelman et al. (2018)
YAP^fl/+^; TAZ^fl/fl^; Prx1^Cre^	Mesenchymal stem cells	BV/TV + Ct. Th +	Ob. N/BS + Oc. N/BS = MS/BS + MAR = BFR/BS +	12-week-old-female	Xiong et al. (2018)
YAP^fl/+^; TAZ^fl/+^; Osx^Cre^YAP^fl/fl^; TAZ^fl/+^; Osx^Cre^ TAZ^fl/fl^; YAP^fl/+^; Osx^Cre^	Young osteoblasts	BV/TV− Ct. Th −	Ob. N/BS − Oc. N/BS + MS/BS = MAR − BFR/BS =	8-week-old male	Kegelman et al. (2018)
YAP^fl/fl^; Ocn^Cre^	Osteoblasts	BV/TV − Ct. Th =	Ob. N/BS − MAR − BFR/BS −	3-month-old male	Pan et al. (2018)
YAP^fl/fl^; TAZ^fl/fl^; DMP1^Cre^	Mature osteoblasts/osteocytes	BV/TV − Ct. Th −	Ob. N/BS − Oc. N/BS + MS/BS − MAR − BFR/BS −	12-week-old-male	Xiong et al. (2018)
YAP^fl/fl^; TAZ^fl/fl^; DMP1(8 kb)^Cre^	Mature osteoblasts/osteocytes	BV/TV − Ct. Th −	Ob. N/BS − Oc. N/BS + MS/BS − MAR − BFR/BS −	Post-natal-day 84	Kegelman et al. (2020)

Synthesis of the bone phenotype observed in different mouse models invalidated for YAP, and/or TAZ, at different stages of differentiation. YAP/TAZ, deletion in osteoprogenitors results in lethality, and later invalidation using Osx, and Ocn or DMP1-Cre decreases bone volume. Obl.S/BS, osteoblast surface, Oc.S/BS, osteoclast surface, MS/BS, mineralized surface/bone surface, MAR, mineral apposition rate, and BFR/BS, bone formation rate/bone surface.

**TABLE 3:** Cartilage phenotype induced by YAP and TAZ modulation.

Genotype or model	YAP/TAZ status	Effect on cartilage and OA	References
Mob1a^fl/fl^; Mob1b^−/−^; Col2a1^CreERT^	YAP/TAZ overexpression from P0	Chondrodysplasia phenotype	Goto et al. (2018)
nls-YAP5SA^KI/+^; Col2a1^Cre^	YAP overexpression	Chondrodysplasia phenotype	Vanyai et al. (2020)
Tg-Col2a1-YAP	YAP overexpression	Protects from OA	Deng et al. (2018a)
Mst1^fl/fl^; Mst2^fl/fl^Col2a1^Cre^	YAP/TAZ overexpression	Protects from OA	Deng et al. (2018b)
Intra-articular injection of YAP siRNA	YAP silencing	Protects from OA	Gong et al. (2019)
Intra-articular injection of YAP inhibitor, Verteporfin	YAP/TAZ silencing	Protects from OA	Zhang et al. (2020)
YAP^fl/fl^; Col2a1^CreERT^	YAP silencing from 8 week-old	Protects from OA	Zhang et al. (2020)
Yap^fl/fl^; Taz^fl/fl^; Col2a1^Cre^	YAP/TAZ silencing	Neonatal lethality	Vanyai et al. (2020)

Synthesis of cartilage phenotypes induced by YAP, or YAP/TAZ, silencing, and upregulation *in vivo* found in the literature. OA, osteoarthritis.

Recently, Vanyai and collaborators demonstrated that YAP/TAZ conditional deletion in chondrocytes from Col2a1 Cre mice (Yap^fl/fl^; Tazfl^/fl^; Col2a1^Cre+^) resulted in neonatal lethality due in part to a cleft palate (Vanyai et al., 2020). The authors highlighted the phenotype inconsistency between *in vitro* and *in vivo* because YAP/TAZ are not required for cell proliferation in the cartilage growth plate *in vivo*. However, this study showed that modulating YAP/TAZ levels does not impair cell proliferation but rather induces skeletal deformities *in vivo* probably via the expression of matrix remodelling genes.

The ubiquitous expression of YAP/TAZ and the tissue-specific regulation of the complex are clues for interactions with several other cell signaling pathways. The Hippo pathway interacts with NF-κB signaling to regulate protease expression and cartilage degradation during osteoarthritis. Conversely, the related effect was investigated by the use of intra-articular injection of YAP siRNA or the YAP inhibitor verteporfin: YAP inhibition protected against osteoarthritis (Gong et al., 2019; Zhang et al., 2020). Indeed, intra-articular injection of verteporfin or deletion of YAP by using YAP^fl/fl^; Col2a1^CreERT^ maintained cartilage homeostasis in osteoarthritic mice (Zhang et al., 2020). Silencing YAP by siRNA inhibited interleukin-1β–induced chondrocyte apoptosis and catabolic gene expression (Gong et al., 2019). Of note, osteoarthritic mice treated with YAP siRNA showed reduced subchondral bone attrition. More studies are needed to fully elucidate and clarify the role of YAP/TAZ in chondrocytes and environmental cells within the joints.

## 5 Conclusion

YAP and TAZ are regulators of bone and cartilage homeostasis that allows for structural and cellular adaptation in response to the microenvironment. YAP/TAZ contribute significantly in bone and cartilage by feeding into the regulation of master orchestrators such as Runx2, Osx, and Sox9. Biomechanical components have a crucial impact on the development of bone and cartilage diseases, so YAP/TAZ are central players for the initiation and progression of the diseases. Therefore, members of YAP/TAZ signaling are potential targets in treating bone and cartilage disorders.
